# Seaweeds as Ingredients to Lower Glycemic Potency of Cereal Foods Synergistically—A Perspective

**DOI:** 10.3390/foods11050714

**Published:** 2022-02-28

**Authors:** Louise Weiwei Lu, Jie-Hua Chen

**Affiliations:** 1 Human Nutrition Unit, School of Biological Sciences, University of Auckland, Auckland 1024, New Zealand; 2 High Value Nutrition, National Science Challenge, Auckland 1010, New Zealand; 3 Institute for Innovative Development of Food Industry, Shenzhen University, Shenzhen 518060, China; 4 Shenzhen Key Laboratory of Marine Microbiome Engineering, Institute for Advanced Study, Shenzhen University, Shenzhen 518060, China

**Keywords:** seaweed, algae, cereal, glycemia

## Abstract

Seaweeds are traditional food ingredients mainly in seaside regions. Modern food science and nutrition researchers have identified seaweed as a source of functional nutrients, such as dietary soluble and insoluble fibers, proteins, omega-3 fatty acids, prebiotic polysaccharides, polyphenols, and carotenoids. Owing to the rich nutrients, seaweeds and seaweed extract can be used as functional ingredients by modifying the nutrients composition to reduce the proportion of available carbohydrates, delaying the gastric emptying time and the absorption rate of glucose by increasing the digesta viscosity, and attenuating the digesting rate by blocking the activity of digestive enzymes. This review presents the concept of using seaweed as unconventional ingredients that can function synergistically to reduce the glycemic potency of cereal products.

## 1. Introduction

The prevalence of type 2 diabetes (T2D) has escalated exponentially from 110 million adults in 1994 to 536.6 million in 2021, and it is estimated to reach 578 million by 2030 and 700 million by 2045 [1,2]. The low- and middle-income countries in total contribute to most adult diabetes cases (414.0 million) in 2021 [3]. When the severe acute respiratory syndrome coronavirus 2 (SARS-CoV-2) emerged in the global pandemic [4], T2D came to be an important risk factor for severe morbidity and mortality related to COVID-19 infection (coronavirus disease-2019) [5,6,7,8,9]. The glycemic management is, therefore, one public priority of the population at a high risk of developing T2D, particularly in Asians who are predisposed to poorer insulin sensitivity and higher postprandial blood glucose excursions compared to Caucasians [10,11,12]. The glycemic index (GI) is a numerical value used to characterize the impact of carbohydrate foods to raise postprandial blood glucose concentration and guide people to choose foods that raise the glucose slowly [13]. The GI value is calculated as the percentage of the area under the 2-h blood glucose response curve (AUC) after consuming food containing 50 g of available carbohydrates in comparison to the AUC generated by 50 g glucose in the same participants [13]. If a food has a GI lower than 56 it is considered to have a low glycemic potency, where a GI between 56–69 is medium, and a GI higher than 69 is high [14]. Adhering to a diet of low glycemic potency (glycemic index (GI) < 56) lowers the acute impact of foods on postprandial glycemic response and is strongly associated with better long-term glycemic management to reduce T2D risk [14].

One challenge regarding glycemic management is that the staple food that accounts for 60% of total daily energy consumption in low- and medium-income countries, such as most of the Asian population, is high GI (GI ≥ 70) refined cereal foods [15,16]. For example, white rice, which has been associated with postprandial hyperglycemia and increased risk of T2D, is the main staple food in the Asian diet [17,18,19]. Although cereals with the lower glycemic potency (i.e., lower GI) is well perceived as a protective dietary factor of the T2D risk [20], people may favor refined cereals with preferred sensory characteristics (i.e., color, odor, texture, and flavor) to the healthy alternative since food habits, culture, and family tradition play a significant role in accepting cereal foods [21]. Replacing refined cereal foods with animal-based foods such as meat and meat products can even further increase the risk of T2D [22] in Asian populations that have already been experiencing a shift towards a modern meat dietary pattern [23,24]. Reducing the glycemic potency of cereal foods becomes an important and practical strategy.

**Figure 1:**
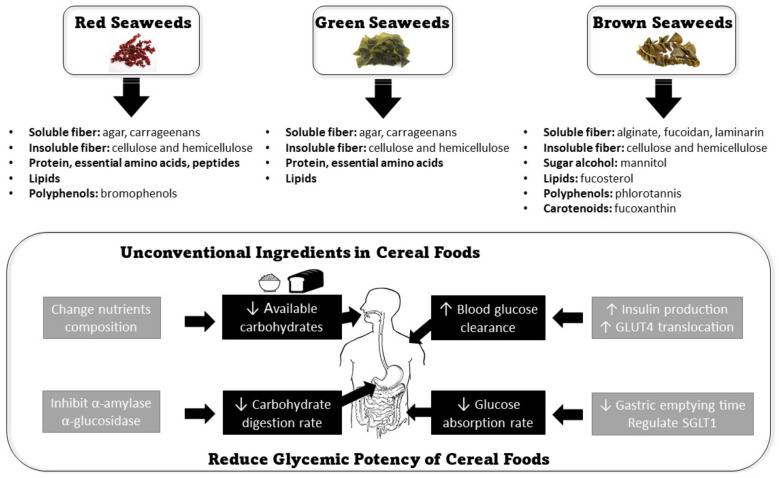
The applications of seaweeds in reducing the glycemic potency of cereal foods.

Current strategies to reduce the glycemic potency of cereal foods are commonly achieved through the following mechanisms: (1) reducing the available carbohydrate, (2) reducing the post-consumption digestion rate, (3) reducing post-digestion glucose absorption rate, and (4) increasing postprandial blood glucose clearance rate [25] (Figure 1).

Seaweeds, which have been used as food ingredients in some coastal regions [26], are a renewable source of high-value nutrients, including dietary fiber, protein, functional fatty acids, prebiotic polysaccharides, polyphenols, carotenoids, and minerals [27]. They can be re-invented as unconventional ingredients in lowering the glycemic potency of cereal foods by modifying the nutrient composition to reduce the energy contribution from available carbohydrates, attenuating the digesting rate by blocking the activity of digestive enzymes, and delaying the gastric emptying time and the glucose absorption rate by increasing the digesta viscosity. In fact, seaweeds have attracted attention from food industries as functional ingredients to fortify the nutritional composition and health-related beneficial properties in bakery, dairy, fish, meat, and vegetable-based food products [28]. According to the Seafood Source report, the number of new food products containing ingredients derived from seaweeds launched in the European market has increased by 147 per cent in four years (from 2011 to 2015) demonstrating Western countries’ growing interest in these substances [28,29].

The previous reviews and perspective articles reviewed the potential biological properties of various seaweeds as a nutritional supplement in the long-term prevention and treatment of diseases, such as anti-cancer, anti-inflammatory, anti-hypertensive, anti-microbial, anti-viral, anti-obesity, and neuroprotective functions [30]. Many studies have focused on the supplementary value of either individual seaweed extract with high-value bioactive nutrients or comparing the nutritional values between different types of seaweeds [30]. Nevertheless, to the authors’ best knowledge, no current article has collectively presented the strategies of using edible seaweeds as added functional ingredients to reduce carbohydrate-rich food’s glycemic potency or glycemic index. As the contents of bioactive compounds vary between seaweed species, reformulating the high GI or glycemic potency of cereal food products does not need to be limited to one seaweed or one nutrient. Instead, multiple seaweed-derived ingredients can work synergistically to combine the different peculiar compositions of various seaweed species. The present article presents the concept of using various seaweeds as innovative ingredients in cereal products, and that can function synergistically to target different mechanisms to reduce the glycemic potency.

In this review, we also summarize published clinical studies that investigated the effect of seaweeds and seaweed extracts on the acute glycemic response in healthy adults, and we review the potential mechanisms that could explain the beneficial role of seaweeds and seaweeds extract on glucose metabolism. The eligible studies include studies that recruited adults (≥18 years old) reported as healthy with a body mass index (BMI) between 18.5 and 40 kg/m^2^, parallel and crossover randomized controlled trials (RCTs) that investigated the acute effect of seaweed species and/or extracts on to up to 3-h postprandial blood or plasma glucose, using placebo as control.

Given that the present article is not a systematic review, we may not have identified some studies and publication bias should be acknowledged. However, both authors conducted the literature search independently using a combination of medical subject heading (MeSH) search terms and free text search terms. The search terms include combinations of “healthy”, “adult”, “normal weight and/or overweight and/or obese”, “seaweed and/or seaweed extract”, “placebo”, “blood glucose and/or plasma glucose and/or serum glucose”, “randomized controlled trials”, and “postprandial”. We searched the publications between January 2000 and January 2022.

## 2. Reducing the Glycemic Potency of Cereal Foods: A Diet Challenge

Cereal foods, which is a group of grains including rice, sorghum, maize, millet, barley, buckwheat, and the staple foods made of flour (e.g., pasta, bread, roti, noodles, etc.), remain a vital high-carbohydrate commodity in nourishing the still increasing world population [15]. Grain processing is a prerequisite for eating cereals, with milling fractionation resulting in the removal of bran fraction together with many compounds beneficial for human metabolism concentrating energy-dense parts into flour. The rapid urbanization in low- and middle-income countries brought dietary change toward an increasing amount of medium and high GI cereal foods [23,31], such as biscuits, cakes, bars, and cereal snacks, which are the carriers of high amounts of added sugar, lipids, and salt [32] (Table 1). GI values of most common cereal foods are in the high GI category (GI ≥ 70) and the medium GI category (GI 56–69) [14].

Incorporating low GI cereal foods in the daily diet should be an important component of glycemic management [14]. Livesey et al. [33] conducted a meta-analysis of 24-prospective cohorts of 7.5 million person-years of follow-up and reported a significant dose-dependent association between glycemic load and the risk of T2D. It is recommended that when the daily carbohydrate intake is between 200–250 g (median value in Western diets), the target GI should be below 40 [33]. When the daily carbohydrate intake exceeds 250 g per day, high GI foods (>70) should be limited to a lower frequency [33]. When the daily carbohydrate intake exceeds 300 g per day (over 60% of energy), an average GI of 33 should be recommended by introducing more low GI foods (GI < 50) [33].

Lowering the glycemic potency (i.e., GI) of cereal foods directly impacts the postprandial blood glucose excursion. The amount of glucose available in the bloodstream mainly depends on several components, including the available carbohydrate content in foods, carbohydrate digestion, hormonal regulation, glucose transport mechanisms and glycolysis [52]. The primary glucose available for the human body comes from the breaking down of the dietary carbohydrate (mostly from starch in cereal foods) by enzymes, including α-amylases and oligossacharidases (e.g., α-glucosidase and sucrase) to monosaccharides (mainly glucose) in mouth and duodenum [52]. In the small intestine, the active transport ensures a one-way flow of glucose D-isomers from the gut to the epithelial cells regardless of glucose gradient via activating sodium Na-glucose transporter 1 (SGLT1) [52]. The absorbed glucose within the capillaries is then transported via mesenteric circulation to target cells for harness energy [52].

The glucose metabolism is closely controlled by pancreatic hormones glucagon and insulin. While the glucagon handles the breakdown of stored liver glycogen and stimulation of gluconeogenesis and ketogenesis, insulin’s roles are to promote glucose uptake by cells, stimulate protein and fat synthesis, and inhibit glucagon activities [52]. Other hormones, including glucose-dependent insulinotropic polypeptide (GIP) and glucagon-like peptide-1 (GLP-1) produced by intestinal L-cell, are also responsible for glucose clearance in blood capillaries, slowing down gastric emptying, suppressing postprandial glucagon production, and increasing glucose-dependent insulin production. After uptake, glucoses undergo glycolytic flux to provide cellular energy and intermediates for energy supply for metabolism in humans [52]. In total, 23% of the glucose is disposed to the brain, 29% to splanchnic tissues (liver and gut), and 25% to insulin-dependent skeletal tissues, while the rest is used by other tissues such as the heart, adipose tissue, and the kidney.

Quality of cereal foods from a nutritional perspective became apparent as people became more aware of the impact of sedentary lifestyles, increased availability of foods with high energy density and high GI and changing eating patterns on health. The interplay between cereal foods quality (nutrition and glycemic potency) and the human digestive system brings about changes in physiology important for the maintenance of healthy homeostasis, particularly glucose homeostasis. Intending to improve the nutritional profile and lower the glycemic potency of cereal foods, research activities around the world emphasized studying physiological mechanisms and factors leading to lower glycemic potency and to the development of technologies suitable for rendering unconventional ingredients to improve the health benefit in cereal food products.

## 3. Seaweed: The Unconventional Potential of the Traditional Food Ingredients in Lowering the Glycemic Impact

The glycemic index can be attenuated by adjusting the nutrient composition to reduce the energy contribution from available carbohydrates, mitigating the digesting rate by blocking the activity of digestive enzymes, and delaying gastric emptying time and glucose absorption rate by increasing the digesta viscosity. Seaweeds, which provide a range of high-value nutrients, such as dietary fiber, protein, functional fatty acids, prebiotic polysaccharides, polyphenols, and carotenoids, can lower glycemic potency via interfering with the mechanisms. In this section, we review the use of seaweeds in modern days and the macro-and micro-nutrients of seaweed that may exhibit glycemic-lowering effects.

### 3.1. Seaweed: A Traditional Food Ingredients in Modern Days

Seaweeds or algae are autotrophic organisms that generate energy through photosynthesis [53]. They are the sea “vegetables” that provide nutrition and energy as feeds to other marine organisms [53]. The seaweeds are classified taxonomically into four main groups according to their pigmentations: red seaweed (*Rhodophyta*), green seaweed (*Chlorophyta*), brown seaweed (*Phaeophyta*), and blue-green seaweed (*Cyanophyta*) [53]. Red seaweed, such as *Corallina officinalis*, *Tenarea tortuosa* (formerly named *Lithophyllum tortuosum*), *Asparagopsis armata*, *Palmaria palmata*, and *Mastocarpus stellatus*, are the most abundant and primitive class. Brown seaweed (phylum *Ochrophyta*, class *Phaeophyceae*) is further divided into two subgroups: kelps mainly in the Western Cantabrians and Iberian Peninsula Atlantic coasts; (*Laminaria hyperborean*, *Laminaria ochroleuca*, and *Saccorhiza polyschides*), and fucales (*Fucus vesiculosus*, *Fucus serratus*, *Himanthalia elongata*, and *Ascophyllum nodosum*) [54,55]. Green seaweed, such as *Ulva lactuca,* is less abundant than the other groups [32]. Currently, only around 200 out of approximately 10,000 species are used as food ingredients [56,57]. Around 10 species are cultivated to provide 30 percent of the 120 million tones (measured in wet weight) of the world aquaculture production in 2019 [57]. In 2021, red seaweed and brown seaweed were ranked the second and the third-largest species groups in global aquaculture, respectively [57].

Red, brown, and green seaweeds have been used as direct food ingredients or processed foods in the coastal regions of Asian countries (e.g., China, Japan, and Korea), few European countries (e.g., Greenland and Iceland), and some South American countries (e.g., Mexico and Chile) [53,58,59]. The average adult in Japan and South Korea consumes around 14.3 g (wet weight) dietary seaweeds per day [60,61]. Amongst the seaweed foods, the most recognized are *Porphyra*/*Pyropia* spp. (Nori), *Laminaria*/*Saccharina* spp. (Kombu), and *Undaria* spp. (Wakame) [61]. Due to the global migration and cultural infiltration and the advancing technologies in food preservation and transportation, more regions have started to consume seaweed as direct food ingredients [53,62,63]. Further, due to the year-round availability, seaweeds have gained much attention as an inexpensive source for plant-derived extract. The seaweeds are commonly extracted to make food hydrocolloids such as agar, carrageenan, and alginates. The growth of consumers’ demands for healthy foods, sustainable food production, and plant-based foods has motivated the research for unconventional use of edible seaweeds beyond the traditional culinary use [64].

From a nutritional standpoint, the nutrients in seaweeds are excellent substances that can improve the carbohydrate quality and lower the glycemic potency of cereal foods. Similar to most terrestrial edible plants, seaweeds are excellent sources of dietary fiber, omega 3 fatty acids, polyphenols, and phytochemicals such as carotenoids [65]. These nutrients have been recognized and endorsed for health benefits and potential development of functional ingredients [57]. These seaweed-derived nutrients may have technological properties that make them viable to be incorporated in food products when the quantity is carefully evaluated to meet the required sensory quality [27,64,65].

Nevertheless, the availability of the nutrients and the nutrient composition of seaweeds growing in the wild are highly variable between seasons [66,67,68]. For example, the total seaweed carbohydrates content in wet weight reaches the highest in the maximum growth periods and in the summer months when photosynthetic activity is the highest [67]. The lipid content is correlated to the temperature change and reaches the highest concentration in winter and spring [69]. The protein content reaches the highest concentration in August [68]. The synthesis of the bioactive compounds generally responds to the intense irradiation and high oxygen concentration that typically happens in February to act against the free radicals and other oxidizing agents [68]. The moisture content of fresh seaweed is very high (up to 94% of the biomass, Table 2), which also varies between seasons in the wild [27]. The advancing seaweed cultivation technologies have solved the problems of nutrient variations in wild-harvested seaweeds [70,71]. For instance, by modifying the abiotic factors, the production of target nutrients can be well controlled [72,73].

### 3.2. Carbohydrates: Dietary Fiber and Polysaccharides

Seaweeds have high amounts of total carbohydrates, ranging between 20–76% of dry weight (DW) depending on the species (Table 2) [27]. The highest total carbohydrate contents are found in species such as *Fucus*, *Ascophyllum*, *Porphyra,* and *Palmaria* [27]. Carbohydrates in seaweeds exist as cell wall structural, storage, and functional mucopolysaccharides [27]. In terms of regulating glycemic potency, seaweed is a good source of carbohydrates alternative, since the majority is available as dietary fibers that cannot be utilized and absorbed by the human body. In fact, seaweed has even higher levels of total dietary fiber than terrestrial plant foods [27,66]. Based on DW, seaweed’s average total dietary fiber content can vary from 36% to 60% (Table 2). The available carbohydrate, such as glucose, mannose, and galactose are in small quantity [27].

Seaweeds contain two main fractions of dietary fibers: around 25% water-soluble fibers [73] and 21.8% insoluble fibers [55]. Soluble fibers, such as agars, alginates, fucoidan, carrageenan, laminararan, and porphyran can form viscous gels in water in the human gastrointestinal tract to promote satiety, improve intestinal microbiota, and slow down the digestion and absorption of other nutrients [27]. Insoluble fibers, including cellulose, hemicelluloses (2–10% and 9% DW, respectively), mannans, and xylan are mainly from cell wall polysaccharides (i.e., the building blocks to support the thallus in water) [27]. They cannot form gels in water or fermented in the large intestine, but can keep water content in the structural matrix to facilitate large intestine transit and increase the fecal mass [27]. The ratio of soluble to insoluble fiber (S/I) in seaweeds varies depending on the type of seaweed and growing conditions, but is generally higher than the values found in terrestrial edible plants [55]. In this review, we are going to scoop the glucose-attenuating effect of the seaweed dietary fibers.

#### 3.2.1. Alginate

Alginate was discovered as a major dietary fiber in brown algae (Phaeophyceae) from the genera *Laminaria*, *Undaria*, and *Hizikia* [27]. It is a glucose polymer that comprises a block-wise monomers α-l-guluronic acid (G residue segments) and β-d-mannuronic acids (M residue segments) to form alternating G-blocks and M-blocks or MG-blocks [103]. The viscosity and gelling forming ability of alginate is contributed by the content of G-residues [104], and play an important role in reducing the post-digestion nutrients absorption and gastrointestinal signaling to control satiation and satiety [105,106]. One unique property of alginate is its ability to form gel in both acid environments (pH < 3.5 at stomach) and form ionic gelation in the presence of divalent cations (such as sodium and calcium) [105]. Now, in both alginates forms, alginic acid (a linear polyuronic acid) and salt (sodium alginates (SA) and calcium alginate Ca-alginate), are widely applied as natural hydrocolloids to thicken aqueous solutions to form desired food formation and quality [107].

Alginate has been reported to decrease the concentration of serum cholesterol [108], prevent the absorption of toxic chemical substances in the gut [109,110], and prevent a postprandial increase of blood glucose and insulin [111]. Previous in vitro research showed that the predominant postprandial blood glucose-suppression effect of calcium alginate is by its ability to inhibit brush border enzyme α-glucosidase [112,113]. In addition, the positive correlation between the viscosity of the starch suspension and the decrease of starch digestion suggests the main suppression effect comes from the increase of viscosity by alginates, which act as a barrier to physically interfere with the contact between α-amylase and starch and between α-glucosidase and maltose [112].

The acute postprandial glycemic potency of alginate-fortified foods and beverages in healthy adults was investigated [114,115,116,117,118,119] (Table 3). The beverages fortified with sodium alginate reported a significant reduction in peak glucose concentration (C_max_) between 6–53.2% [114,115,116,119]. Fortification with higher sodium alginate (8.13 g) more significantly attenuated the C_max_ and postprandial peak insulin than the same beverage with lower sodium alginate (4.06 g) [115]. Compared to a similar quantity of total dietary fiber, sodium alginate exhibited a significant glucose-lowering effect [119]. A pre-load of beverage fortified with sodium alginate extract (15 g) also attenuated the postprandial glucose excursion after a subsequent meal by 40% [116]. The significant reduction of C_max_ and incremental area under the glucose excursion curve (iAUC_gluose_) was also reported for sodium alginate fortified solid food (crispy bar containing 50 g available carbohydrate) [117] (*p* < 0.05). Kato et al. [118] found that both 5% (3.2 g) and 8% (5.0 g) calcium alginate in mixed meals significantly reduced the C_max_ and iAUC_gluose_ (*p* < 0.05) in healthy subjects. The results from acute clinical trials strongly support the potential of using alginate salt (sodium alginate and calcium alginate) to lower the glycemic potency of cereal food.

#### 3.2.2. Fucoidan

Fucoidan is a complex sulphated polysaccharide found mainly in the cell walls of brown seaweeds [120]. It is primarily composed of a pentose sugar backbone comprising of repeating hexose deoxy sugar L-fucose residues that are linked by α-(1–3), α-(1–3)-α-(1–4), and in some minor cases by α-(1–3)-α-(1–2) linkages with sulphate groups presented in C-2, C-3, and C-4 positions [120]. It also contains some other neutral monosaccharides, including glucose, galactose, xylose, mannose, and rhamnose [121,122] and may contain acetate and uronic acids [123].

The monosaccharide composition, the degree of sulphation, and molecular weight of fucoidan highly differ between species and within species between different harvesting seasons [124,125] and is linked directly to its bioactivity [126,127]. The most bioactive fraction of fucoidan is that it contains mainly fucose, variable proportions of galactose, and a high-sulfate low-uronic acid proportion; whereas the low bioactive fraction contains other monosaccharides apart from fucose and a low-sulfate high-uronic acid proportion [128]. Fucoidan’s size varies from <10 kDa (low molecular weight fucoidan (LMWF)), 10–10,000 kDa (medium molecular weight fucoidan (MMWF)), to >10,000 kDa (high molecular weight fucoidan (HMWF)) [123,129]. High molecular weight may cause low cell membrane permeability, low bioavailability, and lower potential clinical efficacy [130]. For example, LMWF (4.58–6.5 kDa) exhibits potential anticoagulant effect, whilst a higher molecular weight >850 kDa lacked certain activity[131,132]. HMWF was reported to have no significant effect on long-term glycemic control in diabetic patents [133]. LMWF, on the other hand, has been reported to impact glycemic control via promoting insulin secretion [127], protecting pancreatic [23], and improving insulin resistance [131,134]. As a result, HMWF depolymerisation to synthesize oligomeric components of smaller molecular weight has been advocated to improve bioactivity and bioaccessibility [129].

In the past 10 years, the antidiabetic potential of fucoidan as an unconventional bio-compound has gained attention with the therapeutic effects at various stages of glucose metabolism. Fucoidan extract from *Undaria pinnatifida*, *Fucus vesiculosus*, and *Ecklonia maxima* has been reported to act as the inhibitor of α-glucosidase [132,135] and implicated as a potential antidiabetic agent [136,137,138]. Fucoidan from *Ascophyllum nodosum* and *Turbinaria ornata* has exhibited an inhibitory effect on α-amylase [136,139]. Fucoidan’s negative charge may help to regulate the SGLT1 to slow down the post-digestion glucose absorption in the intestinal lumen [125]. Fucoidan extracts from *Cucumaria frondose* [131] and *Acaudina molpadioides* [140] have been reported to increase insulin production by activating the PI3K/PKB pathway and increase the blood glucose uptake by muscle and fat tissue via activating glucose transporter 4 (GLUT4) translocation. It has been implicated as an inhibitor of dipeptidyl peptidase IV (DPP IV), which is an enzyme responsible for the rapid degradation of incretin hormones such as GLP-1 [141,142], to increase insulin production [143]. In addition to the acute effect, HMWF has been reported to improve the bowel movements (i.e., stool frequency) in T2D patients [96], which may assist the long-term T2D management. However, a pre-load of low dose (0.5 g) and high dose (2.0 g) fucoidan extract did not report a significant hypoglycemic effect when compared to a placebo (cellulose) in healthy adults [144]. Again, no significant effect on postprandial glycemia was found in adults who are overweight or obese otherwise healthy [145] (Table 3). The inconsistency could be a result of the inclusion of low bioactivity and bioaccessibility of fucoidan as an intervention. Since the inadequate data on molecular weight and degree of sulphation, it is difficult to associate the clinical efficacy to the structural and chemical composition.

#### 3.2.3. Other Dietary Fibers and Polysaccharides

Besides the two soluble fibers, alginate and fucoidan, which stand out for the acute hypoglycemic effect, other dietary fibers are directly and indirectly associated with postprandial blood glucose management [55]. Laminarin, a class of low-molecular-weight (5 kDa) storage β-glucans found mainly in brown seaweeds *Laminaria*, *Undaria pinnatifida*, *Fucus vesiculosus*, and *Saccharina* species (up to 35% DW) has multiple (1,3)-β-D-glucan units comprising (1,3)-D-glucopyranose residues with 6-O-branching in the primary chain and (1,6)-links between chains [146]. Depending on the level of branching, it can be water-soluble or insoluble. Laminarin has many reported biofunctional activities, including blocking starch digestion, activating macrophages leading to immunostimulatory, anti-inflammatory, anti-tumor, and wound-healing activities [147]. Agar (100–30,000 kDa), which commonly presents in red seaweeds *Gracilaria* and *Gelidium*, is the first registered hydrocolloid in Europe (registration number E406) [148]. It contains around 40% of agaro-oligosaccharides, which has been reported to possess a potential activity against α-glucosidase [149], suppressing the generation of pro-inflammatory cytokines and the activity of the pro-oxidative enzyme that associated with producing nitric oxide [150]. Ulvan, a water-soluble sulphated polysaccharide from the green seaweed *Ulva* and *Enteromorpha*, comprises mainly rhamnose, uronic acid, and xylose [27]. A systematic review has discussed its function in anti-oxidant and anti-hyperlipidemic [151], which may assist the long-term glycemic management. Carrageenan (193–324 kDa), commonly obtained from red seaweeds of *Eucheuma* and *Kappaphycus*, are found in three major forms, κ- carrageenan and ι-carrageenan that have gelatinization properties, and a thickening substance λ-carrageenan [55]. Carrageenan gel has been reported to exhibit a range of pharmacological applications, including antiviral properties, by blocking the transmission of HIV [152] and STD viruses [153], anticoagulant activity by inhibiting platelet aggregation [154]. However, since carrageenan has been reported to cause reproducible inflammatory reactions and lead to glucose intolerance [155], it is not recommended as an unconventional food ingredient for people at higher risk of dysglycemia. Mannitol is a sugar alcohol corresponding to mannose found in brown seaweeds *Laminaria* and *Ecklonia*. It is commonly used to replace sucrose in sugar-free compound coating due to its sweet taste [27].

### 3.3. Protein

Total protein content varies significantly between species and harvest seasons. It is generally low in brown seaweed (5–24% DW) and high in red and green seaweeds (10–47% DW) [159]. (Table 2) The content of proteins, peptides, and amino acids of red and green seaweeds reaches the maximum in summer [75,159]. Total protein can represent up to 44% DW in *Porphyra tenera* (nori) and *Ulva* spp. [84], which is comparable to high-protein vegetables such as soy [110]. The maximum content in brown seaweeds is found in *Undaria* in spring, which is around 24% DW [160] followed by *Laminaria* and *Sargassum* (around 20% DW) [90] and *Fucus* (12.99% DW) [74]. Most seaweed species are a rich sources of all essential amino acids at the recommended level by WHO [27]. Although some amino acids, including threonine, lysine, tryptophan, sulphur amino acids (cysteine and methionine), and histidine are limited in algal proteins [66], the levels are still higher than those in terrestrial edible plants [27].

The functional amino acids present in seaweeds have shown key biological activities [66]. Peptides extracted from red seaweed *Palmaria palmate* exhibited significantly higher DPP IV inhibition capacity (IC50 > 200 µM) than the natural origin peptides from edible terrestrial plants, collagen, and milk protein [161]. The collected data suggest the potential for increasing insulin production to improve postprandial glucose clearance [161].

### 3.4. Lipids

Seaweeds contain a lower lipid quantity (1–4.5% DW) than other marine organisms [110]. (Table 2) The contribution of seaweed lipids as a food energy source appears to be low than terrestrial oil-rich edible plants such as nuts and soybeans [162]. However, the proportion of long-chain polyunsaturated fatty acids (PUFAs) in seaweeds is higher than in terrestrial edible plants [162]. Particularly, the eicosapentaenoic acid (EPA) and docosahexaenoic acid (DHA) that belong to the omega-3 fatty acids family are linked to a range of biochemically and physiologically important functions [162]. Red seaweeds contain a higher quantity of EPA, palmitic acid, oleic acid, and arachidonic acid; brown seaweeds contain higher oleic acid, linoleic acid, and α-linolenic acid but low EPA; green seaweeds have a greater quantity of linoleic acid and α-linolenic, palmitic, oleic, and DHA [163]. Both red and brown algae are good sources of omega-3 and omega-6 fatty acids [164]. The amounts and concentrations of these PUFAs vary depending on environmental temperature, with lower temperatures favoring their production. The biological benefit of EPA and DHA on hyperlipidemia, blood pressure, cardiac function, vascular reactivity, endothelial function, and anti-inflammation have been extensively studied [162,165].

Furthermore, the prominent lipid in seaweed oil, phospholipids (62–78% phosphatidylcholine and 10–23% phosphatidylglycerol of the total lipid), has shown greater resistance to oxidation and a higher bioavailability [166]. Phospholipids in foods act as natural emulsifiers, aiding and easing the digestion and absorption of fatty acids, cholesterol, and other lipophilic nutrients [166]. Therefore, seaweed oils have many advantages over plant and fish oils as unconventional ingredients in that they are much more resistant to deterioration (rancidity) [166]. Fucosterol, another prominent sterol found in brown seaweed, has been shown to exert a high affinity of inhibitory effect on α-amylase [167] and α-glucosidase, and promote the postprandial glucose clearance by suppressing the expression of protein-tyrosine phosphatase 1B (PTP1B which inversely regulates insulin signalling) [168,169].

### 3.5. Polyphenols

Polyphenols are not abundant in seaweeds. Compared to red and green seaweed (only <1% DW) [82], brown seaweeds have relatively higher levels of polyphenols (up to 4 g GAE/100 g DW) [27]. One of the main polyphenols in brown seaweeds, phlorotannis, is derived from the polymerization of several phloroglucinol units (1,3,5-trihydroxybenzene), has been reported to inhibit α-glucosidase and α-amylase activities [170]. The inhibitory effect of phlorotannis can be noncompetitive (phlorofurofucoeckol-A, 7-phloroeckol, and dioxinodehydroeckol) and competitive (dieckol and eckol) [171]. Bromophenols, found in both red seaweed (*Rhodymeniaceae*) and brown seaweed (*Leathesia nana*) also exhibit the α-glucosidase inhibitory effect [172]. Furthermore, both phlorotannins and bromophenols can increase the insulin-mediated glucose uptake by skeletal muscles by inhibiting the activity of PTP1B, which regulates the leptin and insulin signaling pathways [172,173]. In vivo studies have reported *Fucus vesiculosus* and *Ascophyllum nodosum* attenuated postprandial blood glucose excursion and dropped insulin peak in rats with diet-induced steatosis [170]. Further studies observed the same hypoglycemic effect in mice with non-alcoholic steatohepatitis (NASH) [170] and rats with diet-induced steatosis [174] after being treated with phlorotannins extract.

### 3.6. Carotenoids

Carotenoids that are synthesized naturally by photosynthetic seaweeds give a range of pigmentations [175,176]. Fucoxanthin is one of the major xanthophyll found abundantly in brown seaweeds such as *Fucus spiralis* (171 mg/kg DW) and *Ascophyllum nodosum* (660 mg/kg DW) [177]. One of the anti-diabetic values of fucoxanthin is supported by its α-glucosidase inhibitory function, which delays the digestion and absorption of glucose [178,179]. Another anti-diabetic mechanism of fucoxanthin is via upregulating the GLUT4 expression in skeletal muscle via the induction of Peroxisome proliferator-activated receptor-γ (PPARγ) coactivator-1α and increasing Akt phosphorylation [172,173]. The animal models (C57BL/6J mice fed in high-fat diet) are reported to have normalized GLUT4 expression and increased uptake of glucose in skeletal muscle [180,181]. The hypoglycemic and hypoinsulinemic effect of fucoxanthin was also reported in diabetic/obese KK-Ay mice [178] and obese KK-Ay mice [179].

## 4. Proposed Strategies for Seaweeds-Derived Functional Ingredients in Glycemic Control

### 4.1. Reducing the Available Carbohydrate

**Figure 2:**
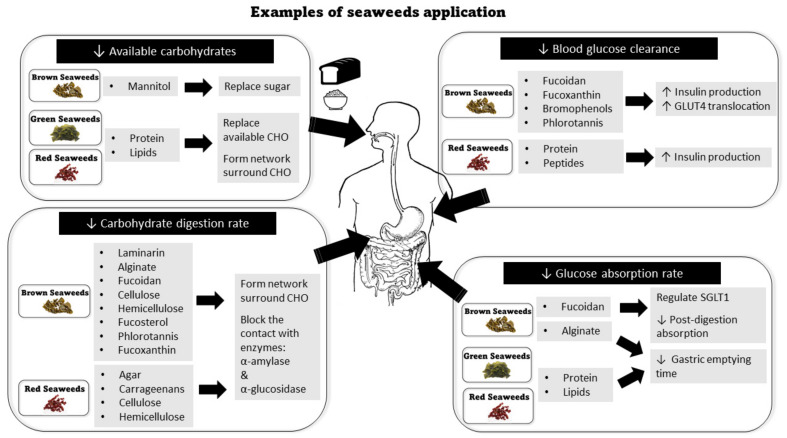
Examples of seaweeds application in various strategies to lower glycemic impact in cereal foods.

Lowering available carbohydrate content can be achieved by replacing the available carbohydrates with other macronutrients derived from seaweeds (Figure 2). For example, mannitol from brown seaweeds *Laminaria* and *Ecklonia* can be used as a replacement for sucrose (i.e., sugar) in sugar-free compound coating without depriving the sweet taste [27]. The full amino acid profile in seaweed proteins [27], particularly the red and green seaweed, which contains up to 47% protein in dry mass [159], makes them perfect candidates to substitute flours in large quantities to reduce the available carbohydrates.

The addition of exogenous proteins to the cereal products can limit the starch availability and digestibility by forming protein—starch interactions network surrounding starch granules, which builds the barrier to digestive enzymatic access [182,183,184]. Further treatment on seaweed protein including hydrolysis [185], low-temperature drying and denaturing [186] could further improve the bioactivity. Similarly, the addition of lipids to cereal products can affect glycemic potency by interacting with starch to form a network surrounding the starch that limits enzymatic accessibility and also starch granule swelling [187]. Under rising temperatures, the exogenous seaweed lipids and starch can form lipid-amylose complexes which have been demonstrated to resist starch digestion [188,189]. A previous study showed olive oil formed a complex with amylose in wheat bread and significantly reduced the postprandial glycemic response as compared to baked bread without oil [190]. Thus, we can expect that the combination of seaweed lipids with high-amylose rice, flour, or other cereal products may also significantly impact glycemic potency.

### 4.2. Reducing Post-Consumption Digestion Rate

Reduction of post-consumption digestion rate can be achieved by blocking the brush border enzyme α-amylase α-glucosidase. Dietary fibers in seaweed are one of the most common ingredients applied to cereal food products to limit starch digestion rate through this mechanism (Figure 2). With different solubility, molecular weight, ionic groups, viscosity, and gelling capacity, different seaweed dietary fibers can provide different functions required to govern the starch digestibility in cereals. Agar and carrageenans from red seaweeds and laminarin (β-glucans) from brown seaweeds (e.g., *Undaria pinnatifida* and *Fucus vesiculosus*) have been used as gelling and thickening agents in food ingredients [55,137,139]. They may form a continuous hydrated network surrounding the starch granules [191] and interact with the starch granule surface to create a barrier for the enzymatic access (α-amylase and α-glucosidase) to starch granules. Thus, the barriers restrict the amylose leaching during the gelatinization [140,186,187]. A similar blocking effect has been found in brown seaweed fibers alginate [112,113] and fucoidan [132,135]. The soluble fiber may further increase the viscosity of digesta [103] after ingestion to reduce the rate of glucose release from digestion in the intestinal phase [192,193]. The rich cellulose and hemicellulose content in all seaweeds may bind α-amylase to inhibit the depolarization of starch, as observed in wheat bran [194].

In addition to dietary fibers, other nutrients found in seaweeds have been identified as α-amylase and α-glucosidase inhibitors and do not affect the viscosity of food products. They include brown seaweed phospholipids, fucosterol [167], two polyphenols, phlorotannis (from brown seaweeds) [170], and bromophenols (from red and brown seaweeds) [172], and fucoxanthin (from brown seaweeds) [180,195]

### 4.3. Reduce the Rate of Post-Digestion Glucose Absorption

The rate of post-digestion glucose absorption can be attenuated by delaying the gastric emptying time. Dietary fibers derived from seaweeds can increase the viscosity of food, which leads to slowing down the gastric emptying rate to slow down the rate of glucose absorption and release into the bloodstream [107,189,191] (Figure 2). For example, during post-ingestion alginate can form solid intragastric gels in digesta to distend the stomach and delay gastric emptying and glucose absorption [115,196]. Fucoidan’s negative charge may help to regulate and manipulate the SGLT1 to slow down the post-digestion absorption of glucose in the intestinal lumen [125].

It is postulated that the addition of lipids and proteins in cereal foods can delay gastric emptying time [197]. Previous acute nutritional human trials demonstrated consuming pancakes with added unsaturated fatty acids (e.g., sunflower oil, olive oil, butter, and medium-chain triglyceride oil) significantly delayed the gastric emptying time and reduced postprandial glycemic response than consuming pancakes without added oil [197]. Co-ingestion of olive oil or grapeseed oil [198], and rapeseed oil [199] ameliorating the GI of cereal foods including rice porridge and bread. Particularly, when both proteins and lipids are co-ingested with the cereal foods, the glycemic impact was more significantly attenuated [199]. In fact, compared to lipids, protein has a more significant effect on slowing down gastric emptying rate [200] by forming protein clots in the acidic stomach [201].

### 4.4. Increase the Rate of Postprandial Glucose Clearance

The blood glucose update can be interfered with by several functional nutrients found in seaweeds (Figure 2). Fucoidan from brown algae *Cucumaria frondose* [131] and *Acaudina molpadioides* [140] may increase blood glucose clearance through two mechanisms: promoting insulin production by activating the PI3K/PKB pathway and inhibiting DPP IV [141,142]; activating GLUT4 translocation [140]. Consuming proteins with carbohydrates in a context meal may also promote insulin secretion [202,203]. The peptides extracted from red seaweed *Palmaria palmate* can significantly inhibit DPP-IV to increase insulin production [160]. Polyphenols, such as phlorotannins and bromophenols can increase the insulin-mediated glucose clearance by skeletal muscle by activating the PI3K/PKB pathway [172,173]. Fucoxanthin has also shown the potential of regulating GLUT4 to increase blood glucose clearance [204].

### 4.5. Future Perspective in Clinical Evidence and Application

The limited evidence from the acute postprandial glycemic study of seaweeds gave mixed results, which require cautious interpretation (Table 3). When consuming *Undaria pinnatifida* (Wakame) with 200 g rice, a significant reduction in postprandial glucose (after 30 min) and iAUC of postprandial glucose response was reported in people with prediabetes [157]. In another study, consuming 70 g *Undaria pinnatifida* (Wakame) with a meal did not significantly change the postprandial glucose excursion in healthy adults, however, a significant reduction in postprandial glucose at 30 min was reported after these healthy adults consumed 70 g *Undaria pinnatifida* sporophylls (Mekabu) with the same meal [158]. However, the preload studies failed to exhibit significant efficacy. The study compared two doses *Fucus vesiculosus* powder (0.5 g and 2.0 g) preload to cellulose as a placebo reported no significant improvement between treatments [144]. Therefore, comparing the soluble fiber rich *Fucus vesiculosus* powder with other fiber that also has attenuating effect on postprandial glycaemia could be the reason for lacking statistically significant difference. Similarly, the combination of *Ascophyllum nodosum* and *Fucus vesiculosus* (0.5 g) preload showed no significant reduction in postprandial glucose but a significant reduction in iAUC of postprandial insulin response [156]. Due to the small sample size and the possible carry-over effect in a cross-over design, it is difficult to interpret and draw conclusions from the limited number of acute clinical trials. Thus, more high-quality clinical trials are required to determine the effective seaweed and cereal foods combination and optimal dose. Moreover, the nutrients content, bioactivity, and bioaccessibility vary significant between seaweeds and within seaweeds between harvesting seasons. The administration should be carefully evaluated to maximize the efficacy of various seaweeds. For example, a combination of protein rich red seaweeds and fiber rich brown seaweeds could add value to the hypoglycemic property.

The common method of incorporating seaweed ingredients is by dispersing them in powder form or the form of extracts as part of the formulation of traditional cereal products [205]. However, a high quantity of soluble fiber may compete with the other ingredients for water absorption and result in a harder food texture [192], which may lower the sensory quality of the cereal products. Therefore, the type and the concentration of soluble fiber used in cereal products should be evaluated carefully to balance the required function without compromising consumer liking. Successful application of seaweeds powder in cereal-based products, such as bread, noodles, cake, cookies, biscuits, and extruded maize. For example, *Himanthalia elongata* was used as a source of dietary fiber to enhance the phytochemical content of breadsticks at doses of 5–15% [205]. To maintain the sensory qualities, the recommended concentration of seaweed-derived products should remain between 0.5–8% in wheat bread, 3–30% in wheat noodles, 5–20% in pasta, 2.5–20% in cakes, 5–60% in biscuits, 3–9% in cookies, and 3.5% in extrudes maize [205].

## 5. Conclusions

Many of the common cereal food in the Asian diet is in the moderate to high GI categories including rice grain, wheat flour, and bakery products that are often made with sugars and refined flour. There is a trend of increasing high GI cereal food consumption in low- and mid-income countries as urbanization accelerate. With the prevalence of diabetes surges, food industries and mindful consumers must incorporate sustainable and functional nutrients from seaweeds as unconventional ingredients into cereal foods to lower the glycemic potency. This review discussed the potential of seaweeds, which have never been a staple food in the Western diet and the diet in inland regions, despite their popularity in a few coastal regions, and the applications based on the synergetic approaches of adding unconventional ingredients.

Seaweeds are a high-quality and nutritious food source by virtue of the high functional dietary fiber and polysaccharides, high content of essential and functional amino acids, high-quality lipids (i.e., omega-3 fatty acids), polyphenols, and carotenoids. Traditionally, seaweeds are used by their technological properties (i.e., gelling, thickening, and stabilizing), such as agar, alginates, and carrageenan as hydrocolloids. Many studies have focused on the supplementary value of either individual seaweeds extract (dietary fiber, omega-3 fatty acids, protein, polyphenols, carotenoids, and minerals) or individual seaweed species in improving the glycemia in people at higher risk of diabetes. To ameliorate acute postprandial blood glucose, reformulate the high GI or glycemic potency of staple food, such as cereal food products, can be achieved by multiple strategies with the addition of multiple seaweed-derived ingredients. As the contents of bioactive and technological compounds vary between seaweed species, the modification does not need to be limited to one seaweed. Instead, multiple seaweed-derived ingredients can work synergistically (i.e., rich functional dietary fibers from brown seaweeds and protein content from red and green algae) to combine the different peculiar compositions of different seaweed species. The strategy’s effectiveness will ultimately depend on how well consumers accept the new textural and sensorial features of the food, whether glycemic impact can be concealed with minimal dietary habit change. The food industry should exploit seaweeds’ nutritional benefits by developing tasty ingredients, reformulated products, and appropriate marketing strategies to reach consumers.

## Figures and Tables

**Table 1:** Glycemic index (GI) of common cereal products.

Food Items	Serving Size (g)	GI Value (Mean ± SEM)	Reference
High-farinaceous food			
White rice	194	96 ± 6.6	[34]
Brown rice	176	66.21 ± 7.78	[35]
Jasmine rice	180.3	78.7 ± 11.6	[36]
Parboiled white rice	259	77 ± 4	[37]
Parboiled brown rice	167	50.1 ± 5.37	[35]
Basmati rice	188.3	50 ± 5.8	[36]
Glutinous rice	109	89 ± 8	[38]
Bario celum (black rice)	50	60.9 ± 7.2	[39]
Beras merah (red rice)	50	78.3 ± 9.9	[39]
White rice porridge	290	98.4 ± 8.1	[40]
Sorghum (coarse)	232	53 ± 2.84	[41]
Sorghum (fine)	252	56 ± 9.83	[41]
Maize (steamed)	164	74.7 ± 6.5	[42]
Millet (steamed)	169	64.4 ± 8.5	[43]
Millet (porridge)	550	93.6 ± 11.3	[43]
Barley powder	67	69.8 ± 6.7	[44]
Processed carbohydrate foods			
Buckwheat noodles	70.2	59.6 ± 13.3	[44]
Wheat noodles	91.5	48.2 ± 4.9	[44]
Wheat pasta	330	72 ± 6.51	[41]
Puffed rice grains	56.2	72.4 ± 6.6	[44]
Rice vermicelli	63.3	56 ± 7	[45]
Rice cakes	93.8	80.7 ± 8.5	[44]
Rice balls	100	96.9 ± 15.1	[44]
Rice dosa	193	76 ± 5	[46]
Rice idli	162	85 ± 4	[46]
Sorghum pasta	330	46 ± 6.47	[41]
Buckwheat jelly	318.5	65.7 ± 11.8	[44]
Bakery foods			
Rice flour muffin	119.4	79.1 ± 6.3	[47]
Rice bread	116.6	73.4 ± 7.6	[44]
White wheat bread	91.4	83 ± 8.8	[48]
Wholemeal wheat bread	128	77 ± 6	[49]
Wheat pancakes	102.8	57 ± 9.7	[44]
White wheat roti	119	64 ± 9.24	[41]
Brown wheat roti	69.44	61 ± 5.77	[50]
Sorghum multigrain roti	119	68 ± 8.63	[41]
Sorghum flakes poha	277	45 ± 5.27	[41]
Wheat flour muffin	126.1	74.4 ± 8.1	[47]
Bagel	104.1	77.4 ± 11.5	[44]
Rye bread	109.4	64.9 ± 18.4	[44]
Corn flour cake	54	85.02 ± 11.21	[51]
Corn flour cookie	71	52.23 ± 6.78	[51]
Corn flour muffin	136.9	74.4 ± 5.4	[47]
Castella cake	114.2	59.9 ± 13.3	[44]
Buckwheat pancakes	169.4	49.9 ± 8.9	[44]
Sorghum biscuits	75	54 ± 6.3	[41]
Processed breakfast cereal			
All-Bran (Kellogg’s Inc., Seol, South Korea)	57.5	51.4 ± 11.1	[44]
Cornflakes (Kellogg’s Inc., Seol, South Korea)	56.2	51.6 ± 10.7	[44]
Rice flakes poha	277	74 ± 4.87	[41]
Wheat biscuits	75	57 ± 11.4	[41]

GI: glycemic index. All GI tests were performed on healthy participants for 2 h using 50 g glucose as the reference food. Serving size (g): the portion size of the food item in each GI test.

**Table 2:** Nutrient composition of some green algae (*chlorophyta*), red algae (*Rhodophyta*), and brown algae (*phaeophyceae*).

Seaweed Species	Moisture (% of WW)	Dietary Fiber		Protein	Lipids		Total Polyphenols	Reference
		Soluble (% DW)	Insoluble (% DW)	Maximum Protein (% DW)	Total (% DW)	EPA (% DW)		
Brown seaweed (*Phaeophyceae*)								
*Ascophyllum nodosum*	67–87	42–64	NA	8.7	3.62	7.24	960 mg PGE/100 g DW	[74,75,76,77]
*Bifurcaria bifurcata*	73	15	23	8.92	6.54	4.09	1990 mg PGE/100 g DW	[76,77,78,79]
*Durvillaea antarctica*	NA	28	44	11.6	0.8	4.95	NA	[80]
*Fucus vesiculosus*	67–82	11	49	12.99	3.75	9.94	1150 mg PGE/100 g DW	[76,77,81,82]
*Laminaria* spp.	73–94	36	10	21	0.8	16.2	NA	[79,83,84,85,86]
*Saccharina latissima*	73–94	17.12	13.11	25.7	0.8	NA	11.1 mg GAE/100 g DW	[79,84,86,87,88]
*Sargassum fusiforme*	61	32.9	16.3	20	1.4	42.4	NA	[81,85,89,90]
*Undaria pinnatifida*	88	30.0	5.3	24	4.5	13.2	4460 mg GAE/100 g DW	[81,85,90,91]
Red seaweed (*Rhyodophyta*)								
*Chondrus crispus*	72–78	22.25	12.04	27.2	2	NA	NA	[74,89,92,93]
*Garateloupiaturuturu*	85	48.1	12.3	22.9	3	NA	NA	[84,94]
*Jania rubens*	NA	NA	NA	11.28	2	NA	56 mg GAE/100 g DW	[95,96]
*Porphyra/Pyropia* spp.	77–91	17.9	16.8	44	1.0	10.4	5530 mg GAE/100 g DW	[84,85,86,87,90,91,97]
*Pterocladiella capillacea*	NA	NA	NA	20.67	2	NA	93 mg GAE/100 g DW	[95,96]
Green seaweed (*Chlorophyta*)								
*Caulerpa lentillifera*	NA	17.21	15.78	9.26	1.11	0.86	NA	[98,99]
*Ulva clathrata*	78–80	21.9	18.7	44	1.5	NA	NA	[80,86,100]
*Ulva lactuca*	78–80	20.53	34.37	44	1.27	0.87	2.86 mg GAE/100 g DW	[80,84,86,101,102]

WW: wet weight; DW: dry weight; GAE: gallic acid equivalents; PGE: phloroglucinol equivalents; NA: not available.

**Table 3:** Human trials of the efficacy of functional seaweed extract on acute postprandial glucose.

First Author, Year (Reference)	Study Design	Subjects	Intervention	Source	Dose	Duration	Effect
El Khoury 2014a [115]	Randomized, placebo-controlled, crossover design study	24 Healthy adults	Low sodium alginate extract vs. placebo chocolate milk	*Laminaria hyperborea*	Study 1: 4.06 g	2 h	↓ C_max_ by 6%
El Khoury 2014b [115]	Randomized, placebo-controlled, crossover design study	24 Healthy adults	High sodium alginate extract vs. low sodium alginate chocolate milk	*Laminaria hyperborea*	Study 2: 8.13 g	2 h	↓ C_max_ by 13% ↓ peak insulin by 46%
Jensen 2012a [116]	Randomized, double-blind, placebo-controlled, 4-way, crossover design study	19 Healthy adults	Sodium alginate extract vs. control preload beverage without sodium alginate	*Laminaria hyperborea, Lessonia trabeculata*	Study 1: 9.9 g	3.5 h	No significant difference
Jensen 2012b [116]	Randomized, double-blind, placebo-controlled, 4-way, crossover design study	20 Healthy adults	Sodium alginate extract vs. control preload beverage without sodium alginate	*Laminaria hyperborea, Lessonia trabeculata*	Study 2: 15.0 g	3.5 h	↓ iAUC _glucose_ by 40%
Huang 2019 [114]	Randomized, double-blind, placebo-controlled, crossover design study	12 Healthy adults	Sodium alginate extract + 172 kcal sugar beverage with soy protein isolate at pH7 vs. control sugar beverage	N/A	0.625 g	2 h	↓ C_max_ by 53.2%
Wolf 2002 [119]	Randomized, double-blind, placebo-controlled, crossover design study	30 Healthy adults	Sodium alginate extract vs. control glucose-based beverage of similar total dietary fiber level	N/A	3.75 g	2 h	↓ iAUC _glucose_ by 75%
Williams 2004 [117]	Randomized, double-blind, placebo-controlled, crossover design study	48 Healthy adults	Sodium alginate extract and guar gum vs. placebo in crispy bar (containing 50g available carbohydrate)	N/A	1.6 g	3 h	↓ C_max_ concentration by 30% ↓ iAUC _glucose_ by 33%
Kato 2018 [118]	Randomized, double-blind, placebo-controlled, crossover design study	15 Healthy adults	Calcium alginate extract vs. control meal without calcium alginate	N/A	Study 1: 3.2 g	2 h	↓ C_max_ by 11% ↓ iAUC _glucose_ by 15%
Kato 2018 [118]	Randomized, double-blind, placebo-controlled, crossover design study	15 Healthy adults	Calcium alginate extract vs. control meal without calcium alginate	N/A	Study 1: 5.0 g	2 h	↓ C_max_ by 15% ↓ iAUC _glucose_ by 21%
Hernández-Corona et al. 2014 [145]	Randomized, double-blind, placebo-controlled, parallel design study	25 overweight/obese adults	Fucoidan extract vs. placebo	N/A	0.5 g	2 h	No significant difference
Murray et al. 2018a [144]	Randomized, double-blind, placebo controlled, crossover design study	38 healthy adults	*Fucus vesiculosus* vs. placebo (cellulose) preload before 50 g of available carbohydrate from white bread	*Fucus vesiculosus*	Study 1: 0.5 g	2 h	No significant difference
Murray et al. 2018b [144]	Randomized, double-blind, placebo controlled, crossover design study	38 healthy adults	*Fucus vesiculosus* vs. placebo (cellulose) preload before 50 g of available carbohydrate from white bread	*Fucus pediculosis*	Study 2: 2.0 g	2 h	No significant difference
Paradis et al. 2011 [156]	Randomized, double-blind, placebo-controlled, crossover design study	23 healthy adults	*Ascophyllum nodosum* + *Fucus vesiculosus* vs. placebo preload before 50 g of available carbohydrate from bread	*Ascophyllum nodosum* + *Fucus vesiculosus*	0.5 g	3 h	↓ iAUC _insulin_ by 12.1% No significant effect on postprandial glucose
Yoshinaga and Mitamura 2019 [157]	Randomized, open-label, 2-period, crossover design	26 adults with pre-diabetes	*Undaria pinnatifida* vs. placebo with 200 g rice	*Undaria pinnatifida*	4.0 g	2 h	↓ Postprandial glucose at 30 min by 7% ↓ iAUC _glucose_ by 8%
Tanemura et al. 2014a [158]	Randomized, placebo (as test meal) controlled, crossover design study	12 healthy adults	*Undaria pinnatifida* vs. control meal with no extract	*Undaria pinnatifida*	70.0 g	3 h	No significant difference
Tanemura et al. 2014b [158]	Randomized, placebo (as test meal) controlled, crossover design study	12 healthy adults	*Undaria pinnatifida* sporophylls vs. control meal with no extract	*Undaria pinnatifida* sporophylls	70.0 g	3 h	↓ Postprandial glucose at 30 min ↓ iAUC glucose 0–30 min

N/A: not available.

## Data Availability

Not applicable.
